# 中国Castleman病诊断与治疗专家共识（2021年版）

**DOI:** 10.3760/cma.j.issn.0253-2727.2021.07.001

**Published:** 2021-07

**Authors:** 中华医学会血液学分会淋巴细胞疾病学组 中国抗癌协会血液肿瘤专业委员会 中国Castleman病协作组

Castleman病（Castleman disease, CD）又称巨大淋巴结病或血管滤泡性淋巴结增生症。近年来国内外对CD的发病机制、诊断和治疗的研究均取得了较大进展[1]–[9]。2018年CD入选国家卫健委发布的《第一批罕见病目录》。2021年中国Castleman病协作组成立。为了规范该病的诊断和治疗，国内相关专家制定了本共识。

一、CD的病理特征

淋巴结病理检查是CD诊断的金标准。病理形态上，CD可分为透明血管型CD（hyaline vascular subtype of CD, HV-CD）、浆细胞型CD（plasma cell subtype of CD, PC-CD）及混合型CD（mixed type of CD）[1]。

1. HV-CD：淋巴结体积通常较大（数厘米至十余厘米），有完整包膜和丰富血供。镜下形态改变主要包括淋巴滤泡增多、生发中心缩小、套细胞区增宽及滤泡间区血管增生。萎缩的生发中心淋巴细胞削减，仅剩余显著的滤泡树突细胞成分，增生的套细胞可呈同心圆状排列或出现“洋葱皮”样外观，部分滤泡内可有多个萎缩的生发中心。滤泡间区淋巴窦消失，多有显著性厚壁小血管增生，且血管壁可出现程度不等的玻璃样变性。部分玻璃样变性的小血管还垂直长入生发中心而形成“棒棒糖”样外观。淋巴结包膜和小梁也多有增厚、增宽伴玻璃样变性。

2. PC-CD：肿大淋巴结的体积通常较小。镜下可见HV-CD样淋巴滤泡，但部分病例或部分病灶的滤泡生发中心萎缩不明显，甚至会出现生发中心增生和扩大，伴有数量显著增多的浆细胞浸润，部分病例可表现为滤泡间区弥漫性、致密的浆细胞增生并完全取代滤泡间区正常结构。多数病例浆细胞形态成熟，无免疫球蛋白轻链限制性表达。

3. 混合型CD：形态特点兼具HV-CD及PC-CD的特征，可理解为两者的过渡形态或组合形式。

CD的病理诊断推荐病变淋巴结完整或部分切除活检，深部或难以切除的病灶亦可行空芯针穿刺活检。免疫组化检测抗体组合（Ⅰ级推荐）应包括CD20、CD79a、CD3、CD38、CD138、Mum-1、kappa、lambda、IgG、IgG4、HHV-8（LANA-1）、CD21（或CD23）、Ki-67等，鉴别诊断抗体组合（Ⅱ级推荐）还可包括CD10、BCL2、BCL6、IgD、cyclin D1、TdT等，可酌情增加EBER原位杂交和IgH重排检测等。

二、CD的临床分型

根据淋巴结受累区域的不同，可将CD分为单中心型CD（unicentric CD, UCD）和多中心型CD（multicentric CD, MCD）[1],[3]。UCD的病理类型以HV-CD多见，但10％～30％的患者为PC-CD或混合型CD[3],[10]；MCD则以PC-CD和混合型CD多见，HV-CD约占20％[9],[11]。

1. UCD：仅有同一淋巴结区域内一个或多个淋巴结受累的CD被定义为UCD。大多数UCD患者无伴随症状，少数UCD患者伴淋巴结压迫症状、全身症状（如发热、盗汗、体重下降、贫血等）或合并副肿瘤天疱疮、闭塞性细支气管炎、血清淀粉样蛋白A型（AA）淀粉样变等[3],[6],[10]。

2. MCD：有多个（≥2个）淋巴结区域受累（淋巴结短径需≥1 cm）的CD为MCD[1]。与UCD不同，除淋巴结肿大外，MCD患者往往还伴有发热、盗汗、乏力、体重下降、贫血、肝功能不全、肾功能不全、容量负荷过多（全身水肿、胸水、腹水等）等全身表现。依据是否感染人类疱疹病毒8型（HHV-8），可将MCD进一步分为HHV-8阳性MCD及HHV-8阴性MCD[1]。HHV-8阴性MCD又可进一步分为无症状性MCD（asymptomatic MCD, aMCD）和特发性MCD（idiopathic MCD, iMCD），前者除淋巴结肿大外，无全身症状和高炎症表现；后者则伴全身症状和（或）脏器损伤表现。iMCD还可进一步分为iMCD-非特指型和iMCD-TAFRO亚型[12]。

CD的临床分型见图1。

**图1:**
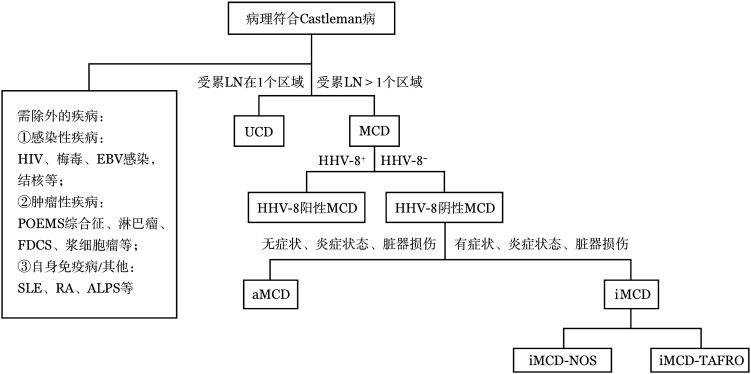
Castleman病的临床分型 HIV：人类免疫缺陷病毒；EBV：EB病毒；FDCS：滤泡树突细胞肉瘤；SLE：系统性红斑狼疮；RA：类风湿关节炎；ALPS：自身免疫性淋巴细胞增生综合征；LN：淋巴结；UCD：单中心型Castleman病；MCD：多中心型Castleman病；HHV-8：人类疱疹病毒-8；aMCD：无症状性多中心型Castleman病；iMCD：特发性多中心型Castleman病；iMCD-NOS：特发性多中心型Castleman病-非特指型；iMCD-TAFRO：特发性多中心型Castleman病-TAFRO综合征

三、CD的诊断流程

有多种疾病（包括恶性肿瘤、感染性疾病及自身免疫性疾病等）也会伴发淋巴结的“Castleman样”病理改变。因此，诊断CD的第一步是排除可能会伴发类似CD淋巴结病理改变的相关疾病[1]，包括（但不限于）感染性疾病（如HIV、梅毒、EB病毒感染，结核等）、肿瘤性疾病（如POEMS综合征、淋巴瘤、滤泡树突细胞肉瘤、浆细胞瘤等）、自身免疫性疾病（如系统性红斑狼疮、类风湿关节炎、自身免疫性淋巴细胞增生综合征等）。

诊断CD的第二步是根据全身查体及影像学检查明确淋巴结受累范围，将患者分型为UCD和MCD。

对于MCD患者，可根据淋巴结组织病理的LANA-1（latency-associated nuclear antigen 1）免疫组化染色和（或）外周血中HHV-8 DNA检测结果判断是否为HHV-8阳性，如果前述两项检测中任一项阳性，诊断为HHV-8阳性MCD；若无HHV-8感染证据，则诊断为HHV-8阴性MCD。

对于HHV-8阴性MCD患者，需进一步明确是否为iMCD。诊断iMCD需要满足以下两条主要标准、至少两条次要标准（其中至少一条是实验室标准），且排除前文所述可能会伴发类似CD淋巴结病理改变的疾病。

主要标准：①淋巴结病理符合CD；②肿大淋巴结（短轴≥1 cm）≥2个淋巴结区域。

次要标准：分为实验室标准和临床标准。实验室标准包括：①C反应蛋白>10 mg/L或红细胞沉降率>20 mm/1 h（女性）或15 mm/1 h（男性）；②贫血（HGB<100 g/L）；③血小板减少（PLT<100×10^9^/L）或增多（PLT>350×10^9^/L）；④血清白蛋白<35 g/L；⑤估算肾小球滤过率（eGFR）<60 ml·min^−1^·1.73 m^−2^或蛋白尿（尿总蛋白>150 mg/24 h或100 mg/L）；⑥血清IgG>17 g/L。临床标准包括：①全身症状：盗汗、发热（>38 °C）、体重下降（6个月下降≥10％）或乏力（影响工具性日常生活活动）；②肝大和（或）脾大；③水肿或浆膜腔积液；④皮肤樱桃血管瘤或紫罗兰样丘疹；⑤淋巴细胞性间质性肺炎。

诊断为iMCD的患者，还应进一步分为iMCD-非特指型和iMCD-TAFRO亚型。诊断iMCD-TAFRO亚型需要符合以下所有主要标准和≥1个次要标准。主要标准：①≥3个TAFRO相关症状（TAFRO相关症状包括：血小板减少、重度水肿、发热、骨髓纤维化、肝脾肿大）；②无明显外周血免疫球蛋白升高；③淋巴结肿大不明显。次要标准：①骨髓中巨核细胞不低；②血清碱性磷酸酶升高但转氨酶升高不明显。

四、CD的预后及危险度分层

UCD的预后良好，5年生存率超过90％[3]，几乎不影响远期生存。但合并副肿瘤天疱疮和闭塞性细支气管炎的UCD患者预后差。

iMCD预后较差，文献报道的5年生存率仅51％～77％[7]。对于iMCD，可借鉴国际Castleman病协作网络（Castleman Disease Collaborative Network，CDCN）的危险度分层体系[2]，符合下述5条标准中2条及以上则考虑重型iMCD，否则为非重型iMCD：①美国东部肿瘤协作组（ECOG）评分≥2分；②eGFR<30 ml/min；③重度水肿和（或）腹水、胸水、心包积液；④HGB≤80 g/L；⑤肺部受累或伴气促的间质性肺炎。

五、CD的治疗

1. 治疗前评估：启动治疗前需对患者进行全面评估，相关检查至少应包括：①症状评估：评价有无发热、疲乏、厌食、体重下降、呼吸困难、皮疹、浆膜腔积液相关症状；评价有无肿瘤压迫相关症状；②影像学检查：颈部、胸部、腹部、盆腔（增强）CT检查或全身PET-CT检查，胸部高分辨CT；③鉴别诊断相关检查：病原学检测（HIV抗体及抗原，EB病毒DNA，梅毒抗体，HHV-8 DNA）、免疫相关检测（抗核抗体谱、类风湿因子、免疫球蛋白定量、IgG4）、M蛋白相关检测（血清蛋白电泳、血尿免疫固定电泳）；④炎症状态及器官损伤评估：血常规、肝肾功能、红细胞沉降率、C反应蛋白、血清白蛋白、乳酸脱氢酶、IL-6、肺功能（通气+弥散）。

2. UCD的治疗：无论UCD患者是否伴有高炎症状态或全身症状，对于有可能完整切除病灶的患者，首选外科手术完整切除病灶[3]。绝大多数UCD患者在病灶完整切除后可达到治愈，极少数病例可能复发。对于复发的病例，可以再次评估病灶的可切除性，若能完整切除，仍然可考虑再次手术切除（图2）[3]。手术不仅能够去除CD病灶，还能够改善相应高炎症状态[10]，改善副肿瘤天疱疮的皮损，改善AA淀粉样变相关症状和膜性肾病。但应注意，手术切除并不能阻止或缓解伴有闭塞性细支气管炎UCD患者的肺部病变，这些患者可能需要肺移植。

对于无法完整手术切除的病例，首先需要评估有无CD相关症状（如压迫相关症状、高炎症状态或副肿瘤天疱疮等）。对于无症状患者，可采用等待观察的策略。对于存在肿块压迫相关症状的患者，可首选利妥昔单抗±糖皮质激素或利妥昔单抗±化疗，对于用药后肿块体积缩小的患者，若可行完整手术切除，则建议手术切除，对于用药后仍难以完整手术切除病灶的患者，可考虑放疗或动脉栓塞治疗。对于伴高炎症状态且病灶难以完整手术切除的UCD患者，可借鉴iMCD治疗方案，采用司妥昔单抗（siltuximab）联合糖皮质激素或沙利度胺-环磷酰胺-泼尼松（TCP方案）等[7],[10]。治疗后应再次评估病灶的可切除性，若药物治疗后病灶缩小且具有可切除性，仍应考虑手术切除[3]。对于药物干预后病灶仍难以切除的患者，若高炎症状态改善，可考虑继续药物治疗并观察肿物变化；若高炎症状态改善不明显，可考虑局部放疗或参考iMCD的其他二线方案（图2）。

**图2:**
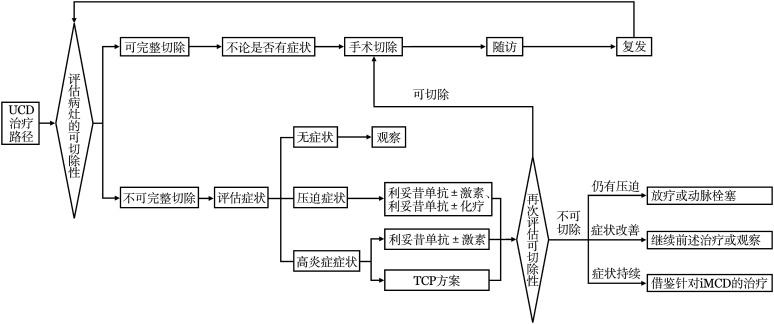
单中心型Castleman病（UCD）的推荐治疗路径 TCP方案：沙利度胺+环磷酰胺+泼尼松；iMCD：特发性多中心型Castleman病；激素：糖皮质激素

3. MCD的治疗：

（1）HHV-8阳性MCD：可以采用以利妥昔单抗为基础的治疗（如利妥昔单抗±脂质体阿霉素/阿霉素±糖皮质激素）[13]。对于同时合并HIV感染的患者，可请相关科室协助制定抗HIV治疗方案。

（2）aMCD：观察随诊。

（3）iMCD：依据CDCN危险度分层定义的“非重型”和“重型”采取不同的治疗策略。由于iMCD的治疗暂无标准方案，无论是对于初治患者还是难治/复发患者，均推荐患者积极参与临床研究。

①非重型iMCD：基于包括目前iMCD治疗领域唯一一项随机双盲对照研究在内的循证医学证据[14]，推荐司妥昔单抗（IL-6单抗）作为非重型iMCD患者的一线治疗方案[2]。该项研究中，司妥昔单抗11 mg/kg每3周1次静脉给药，34％的患者获得了持续肿瘤及症状缓解。其他一线治疗方案包括TCP方案和以利妥昔单抗为基础的治疗[2],[7],[15]。一项前瞻性Ⅱ期临床研究显示，采用TCP方案的患者，48％获得了持续肿瘤及症状缓解[7]。利妥昔单抗虽暂无前瞻性循证证据，但根据病例报告和回顾性研究的数据，亦推荐其作为非重型iMCD的一线治疗方案。对前述某种一线治疗方案疗效不佳或疾病进展的患者可以考虑包括硼替佐米、西罗莫司、来那度胺等药物的单药或联合治疗[2]。单纯糖皮质激素能够改善患者高炎症相关症状，可与前述治疗联合应用（如在司妥昔单抗的基础上，泼尼松1 mg·kg^−1^·d^−1^起始，4～8周后逐渐减量并停用，有效的患者长期使用司妥昔单抗治疗），但不推荐单用糖皮质激素治疗iMCD。

**推荐的一线方案：**

a. 司妥昔单抗±泼尼松：司妥昔单抗11 mg/kg每3周1次静脉给药，治疗有效患者长期用药，直至疾病进展或不耐受；泼尼松1 mg·kg^−1^·d^−1^起始，4～8周后逐渐减量并停用。

b. TCP方案：沙利度胺100 mg每日1次口服，环磷酰胺300 mg/m^2^每周1次口服，泼尼松1 mg/kg每周2次口服，治疗有效患者用药满1年后改为沙利度胺单药维持治疗1年。

c. R-CVP方案：每3～4周为1个疗程，利妥昔单抗375 mg/m^2^，第1天，静脉输注；环磷酰胺750 mg/m^2^，第1天，静脉输注；长春新碱1.4 mg/m^2^（总剂量不超过2 mg），第1天，静脉输注；泼尼松100 mg/d，第1～5天，口服，4～6个疗程。

d. 利妥昔单抗±泼尼松：每4周为1个疗程，利妥昔单抗375 mg/m^2^，第1天，静脉输注；泼尼松1 mg·kg^−1^·d^−1^第1～7天，0.5 mg·kg^−1^·d^−1^第8～14天，口服。

**推荐的二线方案：**

a. 未应用过的上述一线治疗方案。

b. BCD方案：每4周为1个疗程，硼替佐米1.3 mg/m^2^，每周1次，皮下注射；环磷酰胺300 mg/m^2^，每周1次，口服；地塞米松40 mg，每周1次，口服。治疗9个疗程后调整为BD方案（硼替佐米1.3 mg/m^2^，每2周1次，皮下注射；地塞米松20 mg，每2周1次，口服）维持治疗一年或直至疾病进展、不耐受。

c. 西罗莫司（mTOR抑制剂）：单药口服，1 mg/d起始（范围1～7 mg/d），目标谷浓度5～15 ng/ml。

d. R^2^方案：每4周为1个疗程，利妥昔单抗375 mg/m^2^，第1天，静脉输注；来那度胺25 mg/d，第1～21天，口服（根据肌酐清除率调整剂量）。

②重型iMCD：此类患者往往存在显著的器官功能不全，甚至会出现“细胞因子风暴”，患者死亡率高，需要更加积极的干预。推荐一线联合应用司妥昔单抗和大剂量糖皮质激素（如甲泼尼龙500 mg/d，静脉用药，3～5 d）[2]，为了迅速起效，有时还需将起始司妥昔单抗调整为每周用药1次，若治疗有效，1个月后调整为每3周用药1次。由于部分患者疾病进展迅速，前述治疗不一定及时起效（或治疗无效），建议密切评估病情变化，若发现初始治疗效果不佳，则及时（如1周）调整为其他二线治疗，如R±CHOP（利妥昔单抗±环磷酰胺，阿霉素，长春新碱，泼尼松）、BCD（硼替佐米+环磷酰胺+地塞米松）、VDT-ACE-R（硼替佐米+地塞米松+沙利度胺+阿霉素+环磷酰胺+依托泊苷+利妥昔单抗）等。值得指出的是，对于无条件使用IL-6靶向治疗的患者，亦可采用上述化疗方案作为一线治疗。

③iMCD-TAFRO：尽管目前有初步数据提示iMCD-TAFRO的发病机制可能与iMCD-非特指型有一定差异，但基于现有证据，仍推荐对此类患者进行上述危险分层后，参考前述“非重型”和“重型”iMCD的治疗策略进行治疗。此外，环孢素对于iMCD-TAFRO有效，尤其是对于改善腹水和血小板降低。日本TAFRO研究组推荐环孢素联合托珠单抗和糖皮质激素治疗TAFRO综合征[2]。

MCD的推荐治疗路径见图3。

**图3:**
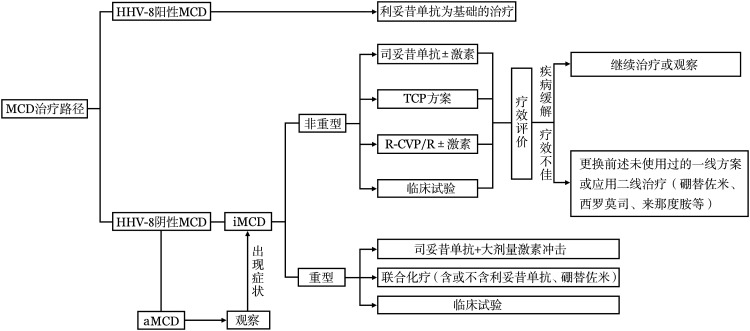
多中心型Castleman病（MCD）的推荐治疗路径 HHV-8：人类疱疹病毒-8；aMCD：无症状性MCD；iMCD：特发性MCD；TCP：沙利度胺+环磷酰胺+泼尼松；R-CVP：利妥昔单抗+环磷酰胺+长春新碱+泼尼松；R：利妥昔单抗；激素：糖皮质激素

六、CD的疗效评估

1. UCD的疗效评价：根据术后1～3个月时的影像学评估手术切除后局部病灶残留情况，之后每年复查影像学，评估有无术后复发。对于存在高炎症状态的UCD患者，可以在治疗后参考iMCD的疗效评估标准评价症状及生化缓解情况。

2. iMCD的疗效评价：iMCD的核心治疗目标是控制高炎症状态，而非淋巴结大小。疗效评价标准推荐采用CDCN 2017年版疗效评估标准（表1）[2]。

**表1:** 特发性多中心型Castleman病的疗效评估标准

整体疗效	生化疗效	淋巴结（根据Cheson标准）	症状改善^f^
CR^a^	CRP、血红蛋白、白蛋白、GFR恢复正常^e^	CR	恢复至基线（发病前）
PR^b^	CRP、血红蛋白、白蛋白、GFR均改善>50％	PR	4个症状（疲乏、厌食、发热、体重下降）均改善，但未恢复至发病前
SD^c^	CRP、血红蛋白、白蛋白、GFR均改善<50％，或恶化<25％	未达PR或CR	4个症状（疲乏、厌食、发热、体重下降）中至少1个症状（但不是所有症状）改善
PD^d^	CRP、血红蛋白、白蛋白、GFR中任一项恶化>25％	增大>25％	≥2次评估提示4个症状（疲乏、厌食、发热、体重下降）中任一症状恶化^g^

注：CR：完全缓解；PR：部分缓解；SD：疾病稳定；PD：疾病进展；CRP：C反应蛋白；GFR：肌酐清除率；^a^指生化疗效、淋巴结、症状改善均达CR；^b^指生化疗效、淋巴结、症状改善均≥PR；^c^指生化疗效、淋巴结、症状改善均未达到PD且不符合PR或CR标准；^d^指生化疗效、淋巴结、症状任一项PD；^e^指CRP≤10 mg/L，HGB≥130 g/L（男）或115 g/L（女），白蛋白≥35 g/L，GFR≥60 ml·min^−1^·1.73 m^−2^；^f^指疲乏或厌食的通用毒性标准（common toxicity criteria，CTC）级别较治疗前下降≥1级，发热较治疗前下降≥1 °C，体重较治疗前增加≥5％；^g^指CTC级别较治疗前恶化≥1级
